# Stock Exchange Pedestrianism

**Published:** 1903-05-09

**Authors:** 


### Stock Exchange Pedestrianism.

The walk to Brighton by members of the London Stock Exchange, which has occupied no small share of the attention of the public for the last month, and which culminated on Friday in a totally unexpected amount of actual achievement, is likely, we think, very much to modify the ordinary popular impressions with regard to the life and habits of the gentlemen concerned. We apprehend that Mr. *Punch*, if his pages, a few weeks ago, had required a representation of a member of the Stock Exchange which should be looked upon as typical, would have sought to supply the want by a figure not only of a certain portliness, but also of a certain rubicundity, such, indeed, as to suggest a greater familiarity with cosy smoking rooms and with well-matured port, than with the attire or the exercises of athleticism. To those behind the scenes it has, of course, long been known that the popular impression was correct, if at all, only in so far as it applied to a few persons; and that the frequently lucrative pursuit in question was by no means incompatible with a warm personal devotion to every description of exercise and of sport. It would, nevertheless, be a surprise even to the most knowing ones to find that 99 starters could be brought to the scratch for an attempt at the very considerable feat of walking more than 50 miles at a stretch, and that of these 78 succeeded in accomplishing the undertaking, 10 of them under 10 hours, and all the rest within 13 hours. The result will lead to quite a new appreciation of the strength and staying powers of the average citizen, and constitutes a very distinct triumph for those who organised and carried out the walk.

It is not surprising, in the circumstances, that the remarkable feat of the members of the Stock Exchange should have stirred up kindred or analogous bodies to emulation; and that we not only hear that Friday's walk is to be made an annual event, but also that bankers, and business men of various denominations, are keenly desirous to prove that they, too, are Englishmen and athletes, who have no intention of leaving all the pedestrian laurels to be reaped by others, while they remain inglorious at their own firesides. And it is in view of this intention, and of its possible or even probable consequences, that we feel compelled, as medical journalists, to take up our parable, even at the risk of being supposed to be out of sympathy with deeds in which our fellow countrymen delight. We cannot allow it to be supposed that the contemplated undertakings will in all cases be free from perils which prudent men, especially those who have given hostages to fortune, should at least hesitate to incur. For the pedestrians who on Friday reached Brighton easily we have nothing but congratulations to offer; but with regard to those who failed to reach it at all, or who arrived distressed or dead beat, the question is not to be so readily dismissed.

It is certain that, as a rule, athleticism is not conducive to longevity, partly, perhaps, on account of the sudden and great variations in condition and habits by which it is apt to be attended. A man who had nothing to do but to get into high condition and to keep himself there, without wasting his strength by over effort at any time, might possibly succeed in the endeavour, although that is a little doubtful; but one who suddenly trains for an emergency, and then, as soon as it has passed, is compelled by the duties of life to allow himself to fall back into merely ordinary health and activity, with a prospect of training again next year for another supreme exertion of all his physical powers, is not pursuing a course which any physiologist would theoretically approve, or which any practical physician would be likely to regard as desirable. The ambition, again, which is stimulated by rewards for "veterans," and which induces men past middle age to return to the scenes of their former contests or victories, is one for which overstrain of heart is more than likely to be the penalty. We fully sympathise with the general admiration for displays of strength and of courage; but, after all, these are relative terms. In physiology, as in physics, it constantly happens that action and reaction are equal; and the subsequent depression of the veteran who "lags superfluous on the stage" is likely to be proportionate to the extent of his effort once more to assert his superiority to competitors. Even to young men, we would counsel prudence in all athletic undertakings. Exercise which is well within the powers of the body is salutary for all, and probably necessary for some; but exercise by which those powers are overstrained is too often not only the precursor, but quite unmistakably the cause, of serious illness or of bodily or mental failure. "Why," inquired Saladin, "should the weak display his inferiority in the presence of the strong?" The question is as pertinent in our own day as it was in that on which it was uttered.

